# Bioactive ZnO Coatings Deposited by MAPLE—An Appropriate Strategy to Produce Efficient Anti-Biofilm Surfaces

**DOI:** 10.3390/molecules21020220

**Published:** 2016-02-16

**Authors:** Alexandra Elena Oprea, Loredana Mihaela Pandel, Ana Maria Dumitrescu, Ecaterina Andronescu, Valentina Grumezescu, Mariana Carmen Chifiriuc, Laurenţiu Mogoantă, Tudor-Adrian Bălşeanu, George Dan Mogoşanu, Gabriel Socol, Alexandru Mihai Grumezescu, Florin Iordache, Horia Maniu, Mariana Chirea, Alina Maria Holban

**Affiliations:** 1 Department of Science and Engineering of Oxide Materials and Nanomaterials, Faculty of Applied Chemistry and Materials Science, University Politehnica of Bucharest,1-7 Polizu Street, Bucharest 011061, Romania; elena_oprea_93@yahoo.co.uk (A.E.O.); loredana.pandel@yahoo.com (L.M.P.); ana_maria_dumitrescu@yahoo.com (A.M.D.); ec_andronescu@yahoo.com (E.A.); valentina_grumezescu@yahoo.com (V.G.); alina_m_h@yahoo.com (A.M.H.); 2 Lasers Department, National Institute for Lasers, Plasma & Materials, P. O. Box MG-36, Magurele 769231, Romania; gabriel.socol@inflpr.ro; 3 Microbiology Immunology Department, Faculty of Biology, University of Bucharest, 1-3 Portocalelor Lane, Sector 5, Bucharest 77206, Romania; carmen_balotescu@yahoo.com; 4 Research Institute of the University of Bucharest, ICUB, Splaiul Independentei 91-95, Bucharest 010271, Romania; 5 Research Center for Microscopic Morphology and Immunology, University of Medicine and Pharmacy of Craiova, Petru Rares Street, No. 2, Craiova 200349, Romania; editor@rjme.ro; 6 Research Center for Clinical and Experimental Medicine, University of Medicine and Pharmacy of Craiova, Petru Rares Street, No. 2, Craiova 200349, Romania; adibalseanu@yahoo.com; 7 Department of Pharmacognosy & Phytotherapy, Faculty of Pharmacy, University of Medicine and Pharmacy of Craiova, Petru Rares Street, No. 2, Craiova 200349, Romania; mogosanu2006@yahoo.com; 8 Flow Cytometry and Cell Therapy Laboratory, Institute of Cellular Biology and Pathology “NicolaeSimionescu” (ICBP), Bucharest 050568, Romania; floriniordache84@yahoo.com (F.I.); horia.maniu@gmail.com (H.M.); 9 Department of Electrical & Electronics Engineering, IDEALAB, Koç University, Rumeli Feneri Yolu, Sariyer, Istanbul 34450, Turkey

**Keywords:** cyclodextrins, zinc oxide, drug delivery, MAPLE, modified surface, biofilm inhibition

## Abstract

Deposition of bioactive coatings composed of zinc oxide, cyclodextrin and cefepime (ZnO/CD/Cfp) was performed by the Matrix Assisted Pulsed Laser Evaporation (MAPLE) technique. The obtained nanostructures were characterized by X-ray diffraction, IR microscopy and scanning electron microscopy. The efficient release of cefepime was correlated with an increased anti-biofilm activity of ZnO/CD/Cfp composites. *In vitro* and *in vivo* tests have revealed a good biocompatibility of ZnO/CD/Cfp coatings, which recommend them as competitive candidates for the development of antimicrobial surfaces with biomedical applications. The release of the fourth generation cephalosporin Cfp in a biologically active form from the ZnO matrix could help preventing the bacterial adhesion and the subsequent colonization and biofilm development on various surfaces, and thus decreasing the risk of biofilm-related infections.

## 1. Introduction

Antibiotics are one of the most important therapeutic discoveries in medical history, and also an essential tool for modern medicine and common procedures, such as transplantation, chemotherapy and orthopedic surgery. Unfortunately, antibiotics have been liable to misuse, leading to the emergence and selection of resistant bacteria. The problem of increasing resistance is even more threatening when considering the very limited number of new antimicrobial agents that are emerging and the ability of microorganisms to form biofilms on natural tissues and implanted medical devices [1]. Extended spectrum β-lactamase and carbapenemase-producing *Enterobacteriaceae* are key Gram-negative pathogens that are involved in serious nosocomial infections. In these Gram-negatives, resistance to all active agents have been described, and clustering of multiple resistance determinants to various classes of antimicrobial agents is a common finding which results in complex multi-drug, extended-drug and pan-drug resistance phenotypes [2,3]. Different clinical studies illustrate that patients infected with resistant strains of these key Gram-negative pathogens have increased mortality, longer hospital stays, and higher hospital costs than those infected by susceptible strains [4].

Zinc oxide (ZnO) has multiple applications in different bio-interface fields, derived from its antimicrobial properties, such as antimicrobial products for topical and systemic administration, antimicrobial coatings, preservative agents for food, active or preservative ingredients for cosmetic and pharmaceutical industries [5,6]. Besides their anti-infective activity, numerous Zn-based materials have been shown to improve wound healing and higher epithelialization rates [7]. For an appropriate evaluation of the promising potential of the nanostructured ZnO used as coatings on medical devices and food packaging with the gradual ion release from the ZnO coatings, which could become toxic over a threshold level, appropriate cytotoxicity assays must be performed [8]. Cyclodextrins are cyclic oligosaccharides represented by glucose derivatives which are approved for being used as pharmaceutical excipients [7,8], revealing a huge potential for the design of future drug-cyclodextrin formulations for oral and parenteral administration. Their molecular structure generates a hydrophilic exterior surface and a nonpolar cavity interior, which can interact with appropriately sized molecules to result in the formation of inclusion complexes [9]. Thus, cyclodextrins can form inclusion complexes with a variety of poorly water-soluble drugs, hydrophobic molecules, improving their water solubility and solution stability, and therefore bio-availability [10,11]. Moreover, cyclodextrins are relatively cheap and could be produced in high amount starting from renewable natural materials, such as starch, by applying eco-friendly technologies, their chemical structure can be easily modified and they are nontoxic in active concentrationsand fully biodegradable [12]. There were already reported some studies proving that material designed for medical uses (*i.e.*, cellulose fabric suitable for medicinal bandages) crosslinked with cyclodextrins and associated with metallic and metal oxide nanoparticles (ZnO, Ag, TiO_2_) exhibited improved resistance to microbial colonization [13]. The aim of this study was to use the MAPLE technique to obtain bioactive coatings based on ZnO and cyclodextrins, incorporating cefepime as a releasable antibiotic and to investigate their antimicrobial properties, cytotoxicity and *in vivo* biodistribution.

## 2. Results

**Figure 1:**
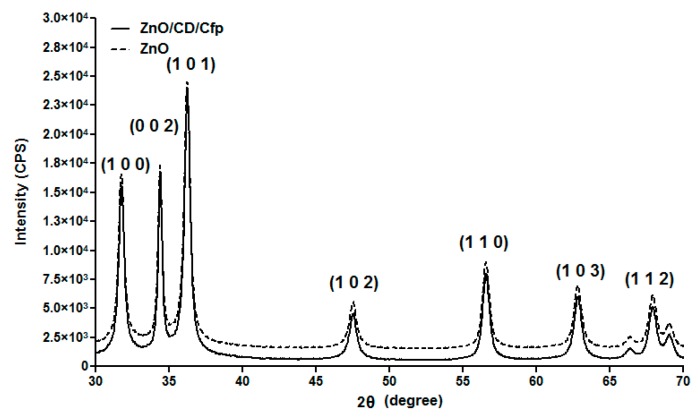
XRD pattern of ZnO and ZnO/CD/Cfp powders.

The prepared ZnO samples were investigated by XRD. As shown in Figure 1, the diffraction peaks correspond to the (1 0 0), (0 0 2), (1 0 1), (1 0 2), (1 1 0), (1 0 3) and (1 1 2) reflection planes and they are indexed to hexagonal ZnO which was matched with JCPDS file No. 036-1451.

**Figure 2:**
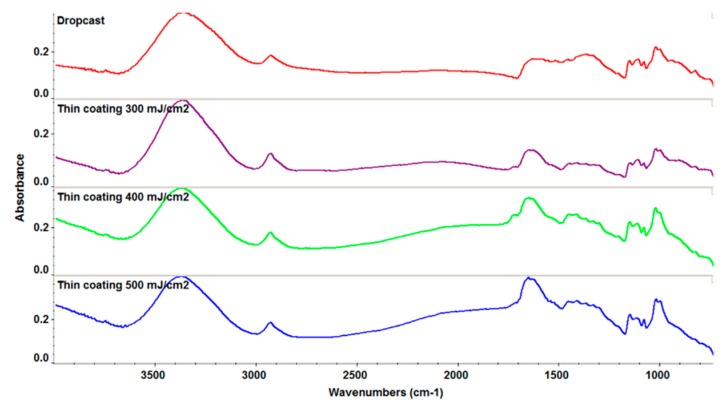
IR spectra of thin films and dropcast for ZnO/CD/Cfp.

A fluence study was performed in order to obtain coatings with a minimum degradation of functional groups and high efficiency of the film growth. IR spectra are plotted in Figure 2.

According to IR analysis it can be concluded that the functional groups were preserved for the laser fluences however the value of F = 500 mJ/cm^2^ was chosen due to the higher deposition rate.

**Figure 3:**
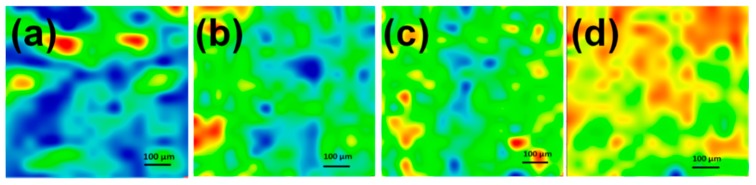
Second derivate IR mappings of dropcast (**a**) and coatings; (**b**) F = 300 mJ/cm^2^; (**c**) F = 400 mJ/cm^2^; (**d**) F = 500 mJ/cm^2^, for ZnO/CD/Cfp.

Infrared microscopy allows the evaluation of an area from the chemical integrity point of view. Figure 3 presents the IR maps built based on 2928 cm^−1^ assigned to CH_2_ from cyclodextrin. The infrared maps of coatings deposited at different fluences were analyzed by comparison with the map of the dropcast. The coatings deposited at F = 300 and 400 mJ/cm^2^ present ~20%–30% of blue spots (that represent the lowest intensity of monitored functional group or degradation of functional group), while the coating deposited at F = 500 mJ/cm^2^ presents <2% blue spots. These results are in good agreement with those provided by infrared spectroscopy. Based on the IR analyses for further experiments it was selected F = 500 mJ/cm^2^ as a compromise between the deposition rate, uniformity of deposition and the stoichiometric transfer.

**Figure 4:**
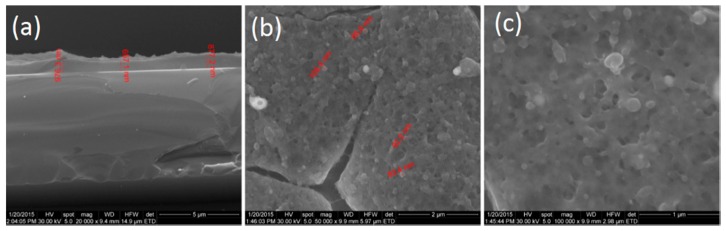
SEM images of ZnO/CD/Cfp coatings (F = 500 mJ/cm^2^): (**a**) cross-section and (**b**,**c**) top view of coatings surface.

Figure 4 shows the cross-section and surface morphology of coatings prepared at F = 500 mJ/cm^2^. From the cross-section morphology image (Figure 4a), it can be seen that the thickness of the prepared thin coating is up to 1 μm. The ZnO/CD/Cfp coatings show a continuous coverage of the substrate with spherical shaped nanocrystals. The ZnO/Cd/Cfp coatings were porous and randomly orientated with thin cracks of micrometer length which were visible on the surface (Figure 4b,c). The average size of particles is up to 100 nm.

**Figure 5:**
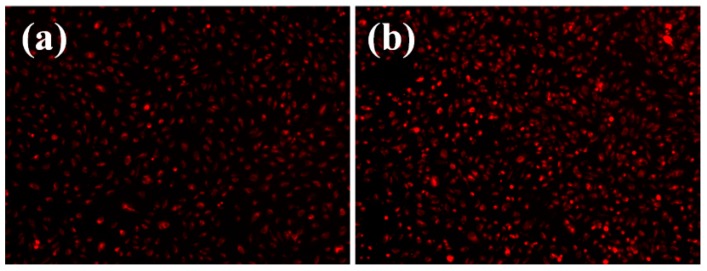
Florescence microscopy images of human endothelial cells (EAhy926 cell line) after three days of growth on (**a**) control and (**b**) coated surfaces (coatings deposited at a laser fluence F = 500 mJ/cm^2^).

**Figure 6:**
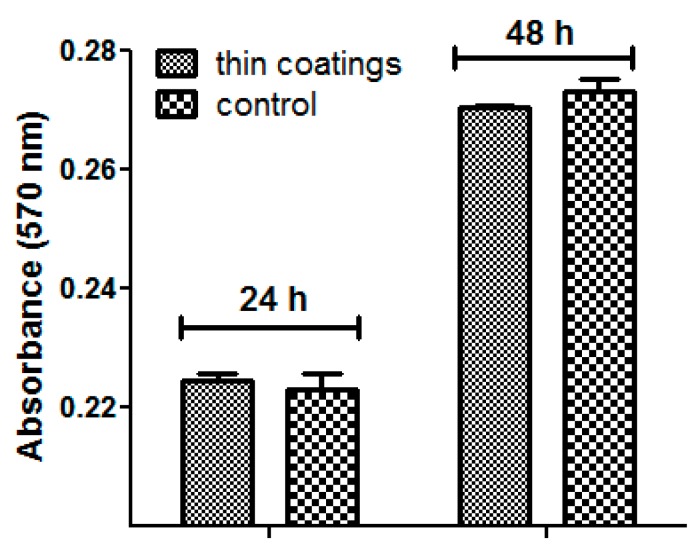
Graphic representation of the MTT assay obtained after the growth of cultured human cells in the presence of the obtained nanomaterials for 24 h and 48 h.

**Figure 7:**
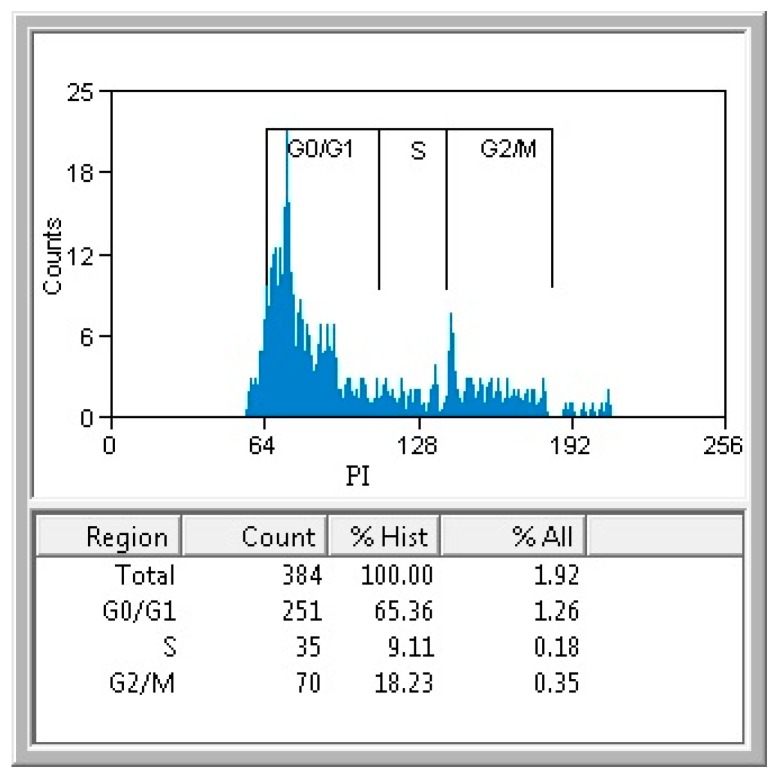
The flow cytometry results of the cell cycle analysis of the cells grown in the presence of tested nanomaterials for 24 h.

*In vitro* cytotoxicity assay revealed that the obtained materials had a good biocompatibility, as demonstrated by the fluorescence microscopy results. Human endothelial cells were perfectly developed in the presence of tested materials, their morphology being similar with control, untreated cells (Figure 5). Furthermore, the quantitative MTT assay revealed no significant differences between the cells grown in the presence of the tested nanomaterials and control cells (Figure 6). The normal growth and development could be confirmed also by the progression of cellular cycle, which reveals that the human cultured cells grown in the presence of the obtained nanomaterials have a typical and normal cell cycle distribution (Figure 7).

### In Vivo Biocompatibility Assay

**Figure 8:**
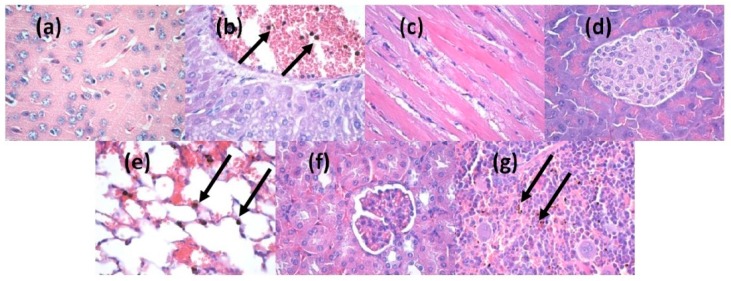
Micrographs of transversal sections through the mice internal organs, treated with ZnO/CD/Cfp for 2 days (Hematoxylin-Eosin staining): (**a**) brain; (**b**) liver; (**c**) myocardium; (**d**) pancreas; (**e**) lung; (**f**) kidney; (**g**) spleen; (×400).

The *in vivo* results demonstrated that at two days after the injection of nanoparticles into the mice veins, the ZnO nanoparticles were absent in the brain, myocardium and pancreas (Figure 8a–c). However, they were found in liver, lung, kidney and spleen (Figure 8d–g). The nanoparticles were revealed as brown-blackish, granular, spherical structures, varying in size, with a diameter of up to 3 μm (arrows marking in Figure 8b or Figure 8e or Figure 8g). In the liver, the nanoparticles were seen in low amounts in blood vessels and Kuppfer cells from the periphery of the sinusoidal capillaries, with a density directly proportional to the size of the sinusoid capillaries of the hepatic parenchyma (Figure 8b). In the lung, the nanoparticles were found primarily in the perivascular macrophages and in the interalveolar septa (Figure 8e), with a density varying depending on cell type. The highest density was observed in the perivascular macrophages and the smallest in the macrophages of the interalveolar septa. Nanoparticles were found also in the intravascular cells of the monocyte-macrophage lineage, probably because of the nanoparticles endocytosis by the macrophage precursor cells from the red bone marrow red, as well as outside monocytes, possibly in platelets. In the kidney (Figure 8f), the nanoparticles have been identified in low amounts in the blood vessels, but not in the remaining renal parenchyma (glomeruli, renal tubules, renal stroma). In the spleen, the nanoparticles were found only in the red pulp, at the level of Billroth cords and sinusoidal capillaries (Figure 8g), while the white pulp revealed hypertrophy, probably due to the stimulation of macrophages after the engulfment of nanoparticles.

**Figure 9:**
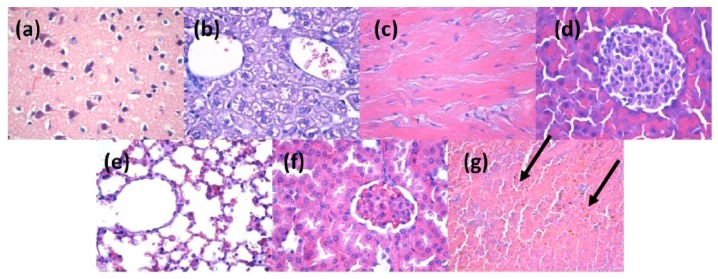
Micrographs of transversal sections through the mice internal organs, treated with ZnO/CD/Cfp for 10 days (Hematoxylin-Eosin staining): (**a**) brain; (**b**) liver; (**c**) myocardium; (**d**) pancreas; (**e**) lung; (**f**) kidney; (**g**) spleen; (×400).

At 10 days after injection, the nanoparticles were absent in brain, liver, myocardium, pancreas, lungs and kidney (Figure 9a–f), being revealed only in the spleen, in the red pulp, at the level of Billroth cords and sinusoidal capillaries, but in a higher concentration than that recorded in samples taken after two days (Figure 9g). The aspect of the white pulp was similar to that registered after two days, with hypertrophy. The density of the nanoparticles varied from one cell to another, some cells exhibiting a larger amount of engulfed nanoparticles.

In the present study, the anti-biofilm activity of the obtained ZnO-cyclodextrin-cefepime was investigated against *E. coli,* which, due to its enzymatic resistance to β-lactam antibiotics, by the production of extended β-lactamases and carbapenemases was included in the list of ESKAPE pathogens [14], containing *E. coli, Staphylococcus aureus, Klebsiella pneumoniae, Acinetobacter baumannii, Pseudomonas aeruginosa* and *Enterococcus* spp., capable of “escaping” from the biocidal action of antibiotics due to resistance mechanisms. 

**Figure 10:**
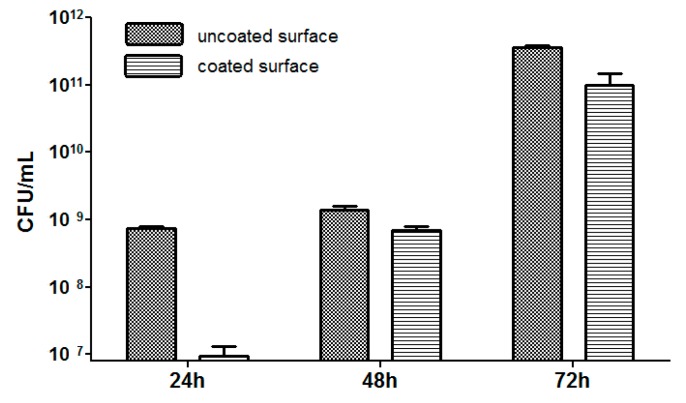
Graphic representation of the biofilm formation results of *E. coli* tested strain, developed on the nanomodified surfaces (F = 500 mJ/cm^2^) for 24, 48 and 72 h.

As shown in Figure 10, the time course study indicated that the growth of *E. coli* strain exhibited a plateau at 24 h and 48 h, followed by a significant increase of the biofilm cellular density at 72 h. The obtained coatings exhibited a very good protective activity against the initial step of *E. coli* biofilm formation, represented by the bacterial adhesion to the substrate and quantified in our study at 24 h and 48 h. The intensity of the inhibitory effect was significantly higher against the 24 h *vs.* the 48 h biofilm, despite the similar number of bacterial cell quantified in the control biofilms at these two time intervals. The tested substratum exhibited also a slight inhibitory activity on the development of the mature biofilm, quantified at 72 h. The time-dependent decrease of the anti-biofilm effect could be due, on one side to the consumption of the antibiotic released from the film, and one the other one, to the fact that the mature biofilm can be protected from the antibiotic action by multiple mechanisms, including the production of the extracellular polymeric matrix and the phenotypic changes induced by the sessile state [15,16].

**Figure 11:**
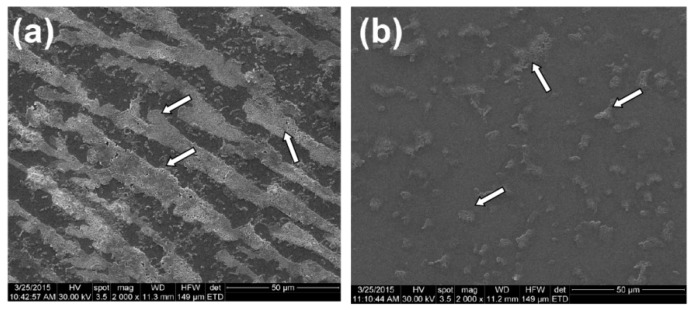
SEM images of biofilmformation of *E. coli* tested strain, developed on the uncoated (**a**) and coated surface (**b**) (F = 500 mJ/cm^2^) for 24 h (specific biofilm aggregates are indicated by white arrows).

The SEM images sustain the results of the quantitative, culture-based assay, revealing the inhibition of the microbial biofilm development on the coated substratum. Therefore, on the uncoated surface we could notice the development of a robust biofilm assuring a good coverage of the examined surface, with a well-represented biofilm matrix, while on the coated surface, only isolated microbial colonies can be observed (Figure 11).

ZnO nanoparticles are frequently used for antibacterial activity against Gram-positive and Gram-negative bacteria strains. The most difficult property to achieve is solubility in water. Bare ZnO nanoparticles are not soluble in water and can easily suffer aggregation. For increasing the solubility and antibacterial activity several methods for the modification of surface chemistry of ZnO nanoparticles have been used. For example, Bai *et al.* [17] have synthesized spherical ZnO nanoparticles of 4 nm and 10 nm average diameters stabilized by dimethyl sulphone with good water solubility and efficient antibacterial activity at low concentrations. Kumar *et al.* [18] have impregnated ZnO nanoparticles with a Cu^2+^ catalyst, the co-catalyst assuring high antibacterial activity. We have utilized cyclodextrin as stabilizing ligand for increasing water solubility of our ZnO nanoparticles. Because cyclodextrins are cyclic oligosaccharides which are hydrophobic inside and hydrophilic outside, they can form complexes with hydrophobic bare ZnO nanoparticles. Thus they can enhance the solubility and bioavailability of our nanoparticles. The observed antibacterial activity for 24 h confirms their high interaction with the bacterial strains due to increased solubility induced by CD chains.

## 3. Materials and Methods

### 3.1. Materials

Zinc nitrate—Zn(NO_3_)_2_·6H_2_O, sodium hydroxide—NaOH, β-cyclodextrin (CD) were purchased from Sigma-Aldrich (Darmstadt, Germany). Cefepime (Cfp) was purchased from a local supplier (Bucharest University Hospital, Bucharest, Romania). All chemicals were of analytical purity and used with no further purification.

### 3.2. Preparation of ZnO/CD/Cfp

ZnO nanoparticles were prepared from a 100 mL solution of Zn(NO_3_)_2_·6H_2_O (3%) and a 100 mL solution of NaOH (3%). These solutions were vigorously stirred at 70 °C and the aqueous basic solution of and β-cyclodextrin (0.5%) was added drop by drop into the Zn(NO_3_)_2_·6H_2_O solution. This dispersion was centrifuged several times in order to remove the secondary products and excess of CD. Then, the powder was baked at 60 °C to obtain dry (ZnO/CD) powder, which was further mixed with 10% cefepime in a grinding mortar in presence of 1 mL of chloroform until complete evaporation of the solvent.

### 3.3. MAPLE Experiment

An amount of 3% (*w*/*v*) colloidal solution was prepared by dispersion of appropriate amount of raw material (ZnO/CD/Cfp = 6:3:1) in DMSO. MAPLE targets were obtained by pouring the solution into a pre-cooled target holder and subsequently immersed in liquid nitrogen for 30 min. MAPLE depositions were carried out using a KrF* laser source (λ = 248 nm and τ_FWHM_ = 25 ns) model COMPexPro 205 (Lambda Physics-Coherent, Ft. Lauderdale, FL, USA) that operated at a laser frequency of 15 Hz. A laser beam homogenizer was used to improve the spatial energy distribution of the laser spot. The laser fluence was within the range of 300–500 mJ/cm^2^ whereas the laser spot area was set to 38 mm^2^. The frozen target was rotated at a rate of 0.4 Hz to avoid the target heating and drilling due to the multiple laser irradiations. All depositions were conducted at room temperature into a background pressure of 1 Pa and a target-substrate separation distance of 5 cm by applying (40,000–60,000) subsequent laser pulses. During the deposition, the target was kept at a temperature of ~173 K by active liquid nitrogen cooling. The coatings were deposited onto double side polished (100) silicon, glass and catheter for physico-chemical analyses and biological assays, respectively. Prior to introduction inside the deposition chamber, the substrates were successively cleaned into an ultrasonic bath with acetone, ethanol and deionized water for 15 min. In order to improve the uniformity of deposition, the substrates were continuously rotated with a frequency of 0.4 Hz. For data comparison, a control set of samples were prepared by drop casting on the double side polished (100) silicon.

### 3.4. Characterization Methods 

Grazing incidence X-ray diffraction (GIXRD) was carried out on an Empyrean Diffractometer using Cu Kα radiation (λ = 1.541874Ǻ) (PANalytical, Almelo, The Netherlands), equipped with a Hybrid monochromator 2 × Ge (2 2 0) for Cu and parallel plate collimator on PIXcel3D detector. The scan was made range of 5°–80° with an incidence angle of 0.5°, a step size of 0.04° and counting time per step on 2θ axis in the of 3 s.

IR mappings were recorded on an iN10 MX FT-IR Microscope (Nicolet, Walthman, MA, USA) with MCT liquid nitrogen cooled detector in the 4000–700 cm^−1^ range. Spectral collection was made in reﬂection mode at 4 cm^−1^ resolution. For each spectrum, 32 scans were co-added and converted to absorbance using OmincPicta software (Thermo Scientiﬁc, Walthman, MA, USA). Approximately 250 spectra were analyzed for each sample. One absorption peak known as being characteristics for the prepared material was selected as spectral marker.

SEM analysis was performed on a FEI electron microscope (FEI Hillsboro, OR, USA), using secondary electron beams with energies of 30 keV on samples covered with a thin gold layer.

### 3.5. Biocompatibility

#### 3.5.1. MTT Test (Using CellTiter 96^®^ Non-Radioactive Cell Proliferation Assay, Promega, Madison, WI, USA)

The MTT assay is a quantitative colorimetric methods that permits the assessment of proliferation, cell viability and cytotoxicity. The method is based on reduction of yellow tetrazolium salt MTT [3-(4,5-dimethylthiazol-2-yl)-2,5-diphenyltetrazolium bromide] to dark blue formazan. The reduction is achieved by the mitochondrial enzymes (especially the succinate dehydrogenase) and is an indicator of the integrity of the cells. Water insoluble formazan can be solubilized with isopropanol, dimethyl sulfoxide or any other organic solvent. The optical density (OD) of formazan is spectrophotometrically evaluated, yielding a function dependent by absorbance and concentration of the cells in culture. The cells were grown in 96-well plates, with a seeding density of 3000 cells/well in different experimental conditions. Then was added 15 mL solution I and incubated at 37 °C for 4 h. Add the solution II and pipette vigorously to solubilize formazan crystals. Incubate 1 h, then pipetted to disperse and remove the bubbles in order not to interfere with the reading. The absorbance was read on a spectrophotometer at 570 nm (TECAN, Männedorf, Switzerland).

#### 3.5.2. CellTracker™ Red CMTPX Assay

The CellTracker™ Red CMTPX fluorescent dye has been designed to freely pass through cell membranes into cells, where it is transformed into cell-impermeant reaction products. CellTracker™ Red CMTPX dye is retained in living cells through several generations. The dye is transferred to daughter cells but not adjacent cells in a population. CellTracker™ Red CMTPX dye is designed to display fluorescence for at least 72 h and the dye exhibits ideal tracking properties: it is stable, nontoxic at working concentrations, well retained in cells, and brightly fluorescent at physiological pH. Red CMTPX was added to the cells at a concentration of 3 μM and incubated for 30 min. The washed cells were analyzed under fluorescence microscope, having an emission spectrum in red (577/602 nm).

### 3.6. Analysis of Cell Cycleby Flow Cytometry Using Propidium Iodide

The treated and control cells were harvested from culture by centrifugation (300 g for 2 min). The supernatant was removed and the cells were resuspended in PBS (phosphate-buffered saline). While mixing, 2 mL ice-cold 70% ethanol was added and samples were incubated on ice for 30 min. Then the cells were spin down and washed in 5 mL of PBS, and resuspended in 400 μL PBS. Then 50 μL of propidium iodide (400 μg/mL) were added and the cells were incubated at 37 °C for 30 min. The samples were analyzed by flow cytometry.

### 3.7. In Vivo Biocompatibility and Biodistribution of Nanostructures

The experimental protocol was applied according with the European Council Directive No. 86/609 (24 November 1986), the European Convention for the Protection of Vertebrate Animals used for Experimental and Other Scientific Purposes (2 December 2005), and the Romanian Parliament Law No. 43 (11 April 2014) on the protection of animals used for scientific purposes. The study was approved by the Ethics Committee of the University of Medicine and Pharmacy of Craiova, Romania (Approval Report No. 118/27.05.2015).

Three months old BALB/c mice were aseptically injected with 100-μL of 1 mg/mL suspension of nanostructures previously sterilized by UV irradiation for 30 min in sterile saline. Intravenous administration was carried out slowly, under general anesthesia (ketamine/xylazine mixture), into the left jugular vein, using a catheter. Reference mice were injected with 100 μL saline suspension. The mice were kept in standard conditions before the organs removal. At 2 days and 10 days after the beginning of the experiment, the animals were euthanized, under general anesthesia, for the sampling of internal organs (brain, liver, myocardium, pancreas, lung, kidney, spleen).

Directly after the sampling, the biological material was washed in PBS to remove blood. Then, the internal organs were fixed in 10% buffered neutral formalin, for 72 h, at room temperature, and processed for routinely histological paraffin-embedding technique [19,20].

### 3.8. In Vitro Biofilm Assay

The microbial strain used in this study, *Escherichia coli* ATCC 8739 was obtained from the American Type Culture Collection (ATCC, Manassas, VA, USA). Fresh glycerol stocks were streaked on nutritive agar plates and colonies were allowed to develop for 24 h at 37 °C. Fresh colonies were used to obtain a bacterial suspension of a 0.5 McFarland (corresponding to 1–3 × 10^8^ CFU/mL) optical density in PBS. For assessing monospecific biofilm formation, 2 mL of nutritive broth were disposed in each well of a 6-wells plate, containing test (coated glass slides) and control (bare glass substrates) samples and seeded with the bacterial inoculum consisting of a volume of 20 μL from the PBS bacterial suspension. After a period of 24 h incubation at 37 °C, the materials containing attached bacteria, were washed with PBS and transferred in a fresh well, containing 2 mL sterile nutritive broth and the incubation continued for another 24 h. The same procedure was repeated at 48 and 72 h, in order to assess the biofilm formation on the materials at different time intervals (24 h, 48 h and 72 h). After each interval, the viable cell count (VCC) method was performed. For this, after each time point, biofilm embedded bacteria cells were detached by vigorous vortexing for 30 s. PBS suspensions containing detached bacteria cells were subjected to serial tenfold dilutions and each dilution was seeded on nutritive agar. Experiments were performed in triplicate and repeated on at least three separate occasions [21].

## 4. Conclusions

The obtained results demonstrate that ZnO/CD coatings deposited by means of the MAPLE protocol exhibited appropriate features for the release of cefepime, as demonstrated by the efficient inhibition of the microbial adherence and development of a mature biofilm on the coated surfaces. These results suggest that the obtained ZnO/CD system could be successfully used as carrier and release systems for the fourth generation cephalosporins, while the MAPLE technique could serve as an appropriate tool for obtaining bioactive coatings, resistant to bacterial colonization. 
